# The effects of aerobic exercise on goal-directed attention and inhibitory control in individuals with high trait anxiety: an EEG study

**DOI:** 10.1186/s40359-025-02376-x

**Published:** 2025-01-30

**Authors:** Mengyang He, Jinxia Guo, Shunyao Yu, Hao Lian, Ruijie Zhan, Rongrong Luo, Zhifei Shi, Zhidong Zhuang, Wenpeng Cai

**Affiliations:** 1 Department of Psychology, School of Sports Medicine, Wuhan Sports University, Wuhan, China; 2 Faculty of Psychology, Naval Medical University, Shanghai, China

**Keywords:** High trait anxiety, Acute aerobic exercise, Top-down attention process, Pd

## Abstract

Anxiety is known to significantly impair cognitive function, particularly attentional control. While exercise has been demonstrated to alleviate these cognitive deficits, the precise neural mechanisms underlying these effects remain poorly understood. This study examines the effects of exercise on attentional control in individuals with high trait anxiety, based on attentional control theory, which suggests that such individuals have reduced top-down attention. Thirty-eight participants were randomly assigned to either an exercise group or a reading group. The exercise group engaged in 30 min of moderate-intensity cycling, while the reading group spent 30 min reading quietly. In Experiment 1, goal-directed attention was assessed using a cue-object paradigm, and in Experiment 2, inhibitory control was evaluated through a visual search task. EEG data indicated that the exercise group exhibited significantly larger Pd components in both experiments, suggesting enhanced attentional focus and improved inhibition of distractors. These findings suggest that aerobic exercise enhances top-down attentional processes, particularly goal-directed attention and distractor inhibition, offering potential as an intervention for individuals with high trait anxiety.

## Introduction

Anxiety is a prevalent mental health condition that affects a substantial portion of the global population, with up to 33.7% of individuals experiencing it at some point in their lives [1, 2]. Individuals with anxiety are often preoccupied with intrusive worries and negative thoughts, which makes it challenging for them to focus on present tasks. This continuous cycle of negative cognitive processing can lead to significant impairments in their cognitive abilities [3, 4]. Trait anxiety refers to an individual’s relatively stable and enduring predisposition to experience anxiety across a variety of situations. Investigating trait anxiety provides critical insights into the underlying mechanisms of anxiety disorders, as it reflects a core feature of chronic anxiety [5]. Specifically, this theory posits that individuals with elevated trait anxiety demonstrate diminished top-down attentional control, a key factor in maintaining optimal cognitive functioning [6–9]. Top-down attentional processing encompasses two essential components: the ability to selectively focus on goal-relevant information and the inhibition of irrelevant or distracting stimuli. These impairments in attentional control may underlie many of the cognitive deficits observed in anxious individuals [10, 11].

A substantial body of evidence supports the view that high trait anxiety significantly impairs top-down attentional processes, particularly in two critical areas: selective attention to goal-relevant information and the inhibition of irrelevant or distracting stimuli. First, individuals with high trait anxiety exhibit a diminished capacity to focus selectively on relevant targets. Empirical studies have shown that these individuals experience deficits in attentional control, as reflected by slower behavioral performance and reduced prefrontal cortex activity during tasks that require target identification [12] and goal-oriented searching [13]. Second, individuals with high trait anxiety face challenges in inhibiting irrelevant stimuli. Research indicates that they are slower to initiate antisaccades away from emotionally neutral stimuli (Derakshan, Ansari, Hansard, Shoker, & Eysenck, 2009; Wieser, Pauli, Alpers, & Mühlberger, 2009) and require more time to detect visual targets presented alongside perceptually salient but emotionally neutral distractors (Moran & Moser, 2015; Moser et al., 2012). Furthermore, these individuals exhibit a reduced capacity to suppress irrelevant information, as evidenced by decreased prefrontal activity and lower neural efficiency [13].

Research has demonstrated that interventions targeting the improvement of attentional control can be particularly beneficial for individuals with anxiety disorders. Such therapeutic approaches aim to equip individuals with more effective cognitive strategies for managing anxiety, leading to enhanced emotional regulation and improved cognitive performance [14]. Among these interventions, recent studies have highlighted the significant benefits of short-term aerobic exercise on attentional control. For instance, Griggs et al. found that participants who engaged in acute aerobic exercise exhibited increased theta power in the brain, accompanied by improved accuracy on tasks requiring attentional control [14]. Similarly, Tsukamoto et al. demonstrated that reported that short-term aerobic exercise significantly enhanced performance on cognitive tasks, particularly those that involve attention and executive control [15]. Moreover, a study examining the impact of exercise intensity during acute intermittent exercise (IE) on executive function and heart rate in twenty male subjects revealed that moderate-intensity exercise optimizes cognitive benefits while minimizing perceived exertion and fatigue [16].

Regular aerobic exercise has been shown to have a significant positive impact on cognitive function, by enhancing key executive processes. It is well-established that exercise not only benefits mental health—by improving mood and alleviating anxiety—but also boosts cognitive functions such as attention and executive control. These effects have been observed in both acute and long-term exercise interventions, highlighting exercise as a promising tool for psychological and cognitive enhancement [17, 18]. Even a single session of moderate-intensity aerobic exercise can enhance cognitive functions like attention, working memory, and executive function in healthy individuals. For instance, Aly and Kojima (2020) found that acute exercise improves neural resources related to perceptual and cognitive processes, as measured by event-related potentials (ERPs) [19]. Similarly, Pontifex et al. (2015) demonstrated that a single bout of exercise can sustain attentional processes, further supporting the immediate cognitive benefits of exercise, particularly for attention and cognitive control [20]. Moreover, this positive effect has also been found in different populations. For instance, research has emphasized the potential of physical exercise, with a particular focus on aerobic activities, as efficacious strategies for diminishing anxiety symptoms among college students [21, 22]. Additionally, a study was designed to investigate the alterations in executive function during and after acute moderate aerobic exercise among adolescents, which likely involved evaluating diverse components of executive function, including working memory, cognitive flexibility, and inhibitory control, at multiple time points throughout and following the exercise session [23]. Furthermore, exercise training can also improve multiple domains of executive function in older adults [24].

Aerobic exercise has been demonstrated to exert a positive influence on cognitive functions, especially top-down attentional control and inhibitory processes, in individuals with high trait anxiety. For instance, Cooper and Tomporowski (2017) found that acute exercise leads to a reduction in attentional bias towards anxiety-related stimuli, particularly in high-anxiety individuals. This indicates that exercise can transiently enhance cognitive control, which is a key aspect of top-down attentional control [25]. Moreover, research has explored how physical activity reduces physiological stress responses and promotes neuroplasticity. By alleviating stress and facilitating neural plasticity, exercise creates a more conducive environment for the brain to optimize top-down attentional control and inhibitory processes, consequently leading to a reduction in anxiety symptoms [26]. In addition, a study explored the impact of regular physical activity on the effect of acute exercise regarding the amygdala’s functional connectivity with the prefrontal cortex, which is closely related to the improvement of inhibitory control and emotional regulation in anxiety regulation. The results showed that habitual physical activity enhances the anxiolytic effects of acute exercise by modulating amygdala connectivity, suggesting a synergistic relationship. This further highlights the importance of physical activity in modulating brain regions associated with top-down attentional control and inhibitory processes ([26]. Furthermore, another relevant study investigated the influence of prefrontal cortex (PFC) stimulation on the amygdala’s response to threat in individuals with high trait anxiety [26]. The findings disclosed that PFC stimulation enhances regulatory control over the amygdala, diminishing its hyperactivity during threat processing. Collectively, these studies highlight aerobic exercise as a potent intervention for alleviating cognitive deficits related to top-down attentional control and inhibitory processes, as well as for enhancing emotional regulation within high-anxiety populations. While there is substantial evidence supporting the cognitive advantages of acute aerobic exercise [27–29], further research is needed to provide direct and comprehensive evidence on whether aerobic exercise specifically enhances top-down attentional control processes relevant to anxiety disorders, as predicted by attentional control theory.

The current study addressed two key research questions by utilizing both a cue-object paradigm and a visual search paradigm. The cue-object paradigm allowed for the objective identification of the target based on cue location, which represents top-down biased selection, actively engaging cognitive processes at a higher level [30, 31]. In contrast, the visual search paradigm, which manipulated the positions of distractors and targets, was employed to dissociate the processing of these elements. This design makes the paradigm particularly well-suited for examining distractor inhibition [32–34]. To select suitable participants, the State-Trait Anxiety Inventory (STAI), a widely used 40-item self-assessment questionnaire, was administered to assess both state and trait anxiety levels [35]. In Experiment 1, participants were tasked with identifying a target based on a preceding cue, reflecting their ability to utilize top-down attentional control. In Experiment 2, participants performed a visual search task where they had to locate a color-singleton target while ignoring distractors, allowing for the assessment of attentional inhibition. To evaluate both target and distractor processing, event-related potential (ERP) components associated with attentional suppression, specifically the Pd component, were analyzed in both experiments (Pd; [36]). We focused on the Pd (distractor positivity) component, as it is specifically linked to attentional control mechanisms, particularly in visual distractor suppression. The Pd component is well-documented for its sensitivity to top-down attentional processes, which are crucial for inhibitory control, especially in individuals with high trait anxiety [25, 37]. Given our focus on how aerobic exercise impacts attentional control, the Pd provided a targeted and relevant neural marker.While other components like N2 and P3 also relate to cognitive control, they reflect broader processes such as conflict monitoring and target detection, which are less directly tied to the specific form of visual distractor inhibition we sought to investigate [38, 39] By focusing on the Pd, we were able to more precisely examine the neural mechanisms most relevant to attentional inhibition in the context of trait anxiety, while minimizing potential confounds from broader cognitive control processes.The study hypothesized that individuals with higher levels of anxiety, due to their impaired attentional control, would exhibit a significantly larger Pd (indicating stronger suppression) following exercise in both tasks. This hypothesis aims to investigate whether exercise-induced improvements in attentional control are evident in anxious individuals, particularly in terms of enhanced target selection and distractor inhibition.

## Methods

### Participants

#### STAI prescreening

In total, 651 students of non-sports majors from Wuhan Sports University was voluntarily recruited for the experiment and prescreened using the Chinese Revised State-Trait Anxiety Questionnaire [40]. Those who scored 55 or higher on the trait anxiety questionnaire were considered to have high trait anxiety and were selected to participate in an EEG experiment. The cutoff score of 55 was chosen based on established thresholds from previous ERP studies investigating anxiety [40, 41]. Additionally, to control for potential confounding effects of daily physical activity on experimental outcomes, participants’ physical activity levels over the preceding week were systematically recorded. To ensure homogeneity across the sample, participants were required to meet three specific inclusion criteria regarding their physical activity during the week prior to the experiment. This study was approved by the Institutional Research Ethics Committee of Wuhan Sports University (2023016).

①High-intensity physical activity of 20 min per day for less than 3 days;

②A total of less than 5 days per week of exercise of all intensities combined and a total force activity level of less than 600 MET-min/w;

③Less than 30 min of moderate intensity activity and less than 5 days of walking per week.

#### EEG test

In Experiment 1, 28 participants were needed based on a priori power analysis with a medium effect size (f = 0.25) and high statistical power (80%) at an alpha level of 0.05. In Experiment2, 24 participants were needed based on a priori power analysis with a medium effect size (f = 0.25) and high statistical power (80%) at an alpha level of 0.05. A total of 80 participants who scored above 55 on the trait anxiety scale were selected, with 40 participants assigned to Experiment 1 and 40 to Experiment 2.To account for potential dropouts and exclusions. 2 participants were excluded due to excessive artifacts in the EEG recordings (artifacts > 40%), leaving 38 participants (29 females and 9 males) for the final analysis of both behavioral and EEG data. The exercise group comprised 18 participants (4 males and 14 females, mean age 20.19 ± 1.21), while the control group consisted of 20 participants (5 males and 15 females, mean age 20.26 ± 0.98). Similarly, in Experiment 2, 3 participants were excluded due to excessive EEG artifacts (artifacts > 40%), resulting in 37 participants being included in the final analysis. The exercise group in Experiment 2 included 18 participants (3 males and 15 females, mean age 19.94 ± 1.43), and the control group comprised 19 participants (4 males and 15 females, mean age 21.26 ± 2.68). Before EEG data collection, participants were asked to complete the State-Trait Anxiety Inventory (STAI) again to ensure they still met the predetermined criteria for high or low anxiety levels. In addition, the Physical Security Status Questionnaire and the International Physical Activity Questionnaire (IPAQ) were administered to monitor participants’ physical activity over the previous week. All participants had normal or corrected-to-normal vision, and none reported a history of neurological or psychiatric disorders. The experiment adhered to ethical guidelines, with all participants providing informed consent prior to participation, and they were compensated for their involvement upon completing the study (See Table 1).

**Table 1:** Scores of the exercise and reading groups on each scale for experiment 1

Group	Trait anxiety score	Baker depression score	Physical activity level (MET-min/W)
Exercise Group	56.35 ± 7.475	13.76 ± 7.287	527.74 ± 15.62
Control group	56.47 ± 5.582	15.32 ± 7.021	538.10 ± 20.65

Note: The independent sample t-test showed that the difference in trait anxiety scores, Baker depression scores, and physical activity water was not significant

#### Intervention

##### Acute aerobic exercise

Subjects in the exercise intervention group perform moderate the intensity [42] of aerobic exercise for 30 min using a German Ergoselect 100 power bike, while wearing a Suunto HR belt to record their heart rate data. The exercise session consisted of three stages: warm-up, main exercise, and cool-down [43, 44]. During the warm-up stage, the subjects pedaled at a speed of 25w to prepare their muscles and cardiovascular system for the main exercise. The main exercise stage lasted for 20 min, during which the subjects were asked to maintain a pedaling speed of 70–80 rpm and to exercise at a moderate intensity. The subject’s heart rate was monitored every two seconds and calculated using the formula THR= (HRmax-HRrest) ×desired intensity + HRrest, where HRmax is the maximum heart rate, HRrest is the resting heart rate, and the desired intensity is based on the ACSM exercise guidelines [45, 46]. The cool-down stage lasted for 5 min and involved gradually reducing the load on the bike to 15w. This allowed the subjects’ heart rate to decrease gradually, reducing the risk of exercise-related injuries, and allowed their muscles to relax.

##### Control group task

Sit quietly in the laboratory to complete a 30-minute free reading task.

#### Stimulation and experimental procedure

##### Experiment 1: Cue-object paradigm

The stimulus is displayed at a fixed distance of 70 cm from the subject, appearing as two gray boxes positioned symmetrically on either side of the screen. Each trial comprises two main components: a clue array and a discrimination array. Initially, an empty screen is presented for 800-1000ms, followed by a 200ms clue array. The clue array includes a central gaze point along with horizontal and vertical gray line segments, with their positions randomly changing within the gray boxes on each side of the screen. Notably, each clue array consistently contains both horizontal and vertical line segments. Subjects are instructed to focus their attention on either the horizontal or vertical line segment, with half of the participants assigned to each condition. Following the clue array, a blank screen is displayed for a duration of 1400 ms to 1600 ms before the discrimination array appears for 200 ms. This array comprises a central gaze point and two solid circles of varying colors, positioned within the left and right boxes respectively. The color of each circle is randomly chosen from red, green, or blue, with equal probability. Additionally, within each block of trials, one of the first three colors is designated as the target color, with the presentation order randomized across the three blocks.A valid cue is defined as a cue for which the indicated location is in precise correspondence with the actual location of the target stimulus. In such a scenario, the cue functions as a predictor of the target’s location, consequently enhancing the attentional engagement at the corresponding site.

An invalid cue designates a scenario in which the cue designates a location that fails to coincide with the actual target location. Such misdirection of attention typically results in a delay or interference in response time, denoting an enhanced necessity for attentional reallocation.

The neutral cue condition entails a cue devoid of any information about the target location. It functions as a baseline, in which the cue bears no relevance to the location of the target and is projected to yield a neutral influence on attention allocation.

**Fig. 1:**
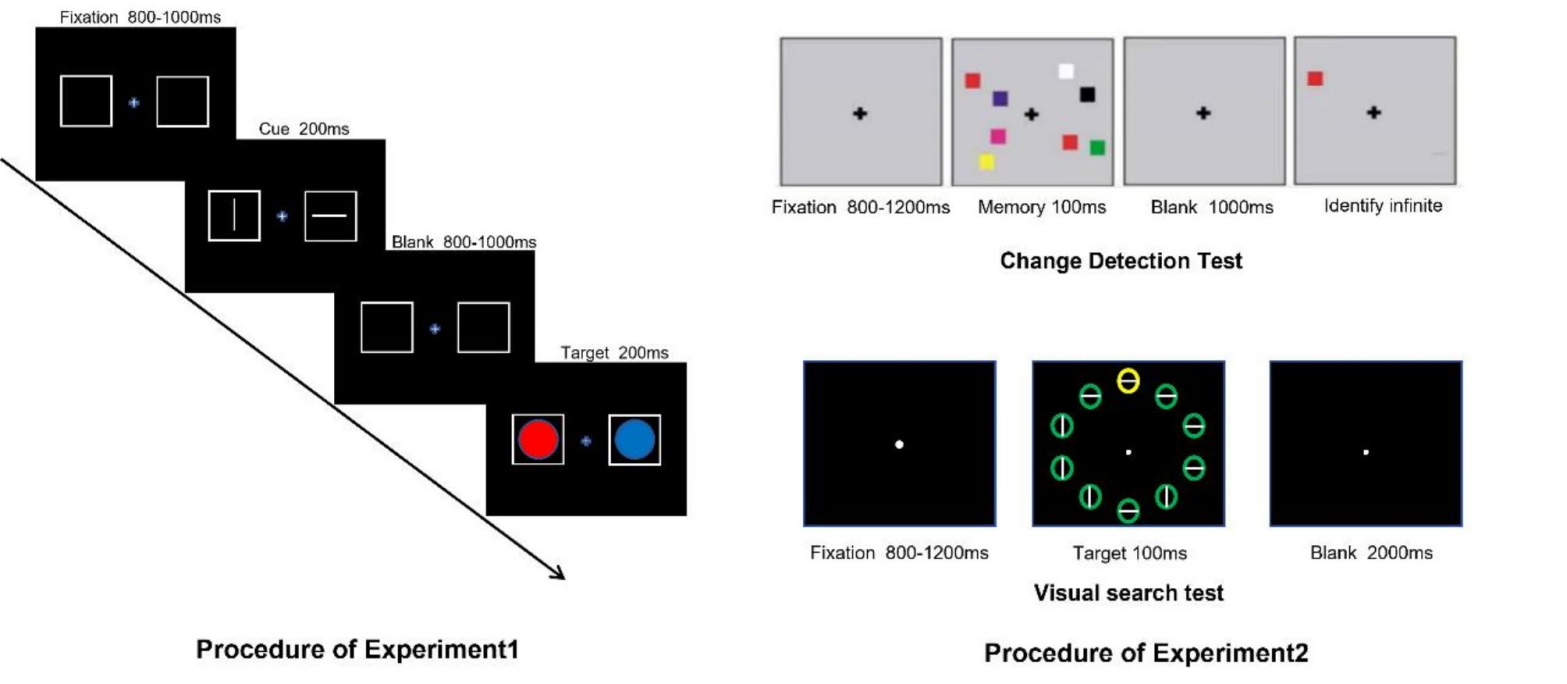
Procedure of experiment

Subjects were asked to indicate whether there was a target color circle in the clue position through key-press reaction, and if there was a target color, press the “Z” key and there was no “M” key. During the whole attention process, the subjects are required to keep their eyes on the central fixation point and respond as quickly as possible on the premise of ensuring the correct rate. The experimental procedure is shown in Fig. 1.

##### Experiment 2

The study comprises two distinct tasks. Before beginning the EEG experiment, all participants underwent a working memory capacity assessment using the Change Detection Paradigm. This assessment was conducted to ensure that there were no significant differences in working memory capacity between the two groups. By confirming comparable working memory abilities, participants were better able to engage in the subsequent visual search task, which served as the basis for EEG data collection. This approach ensured that any observed differences in EEG recordings could not be attributed to disparities in working memory capacity.

#### Change detection paradigm

The Change Detection paradigm consisted of a stimulus memory array and a detection array. In the stimulus memory array, a fixation cross would appear in the center of the screen for 1000 ms, then several squares ranging from 4,6, and 8 (the number of squares presented on the left and right sides of the eye point was equal) presented for 150 ms, the participants were required to remember the color and location of each square as much as possible. After 1000ms, a square appears on the screen, the participants were instructed to judge whether the color of this square is the same as that of the square in the same position in the previous memory array by pressing different keys in the keyboard (“F” for the same, “J” for different). At the end of the reaction, the test array is presented until the subject responds. All colors are presented randomly, and no more than 2 squares of the same color will appear on the same side of the memory array, consistent and inconsistent trials were 50% each, with 5 block and block 24 trial each. The duration of the experiment is about 10 min. The flow of the experiment is shown in Fig. 1.

The change perception task used Psychopy3 software to present the stimuli. The stimuli used for testing were 3.4° ×3.4°squares, and all stimuli were presented within a rectangular area of 4°×7.3°in size at a horizontal distance of 3° from the central cross gaze point. The screen background was gray (192,192,192), colors included 9, red (255,0,0) Green (0,255,255) Blue (0,0,255) Green (0,255,0) Yellow (255,255,0) purple (255,0,255) orange (255,128,0) black (0,0,0) white (255,255,255) the minimum distance between squares is 2° (center to center).

### Visual search test

All experimental stimuli were presented on a “17” DELL display (1600 × 900 resolution, 60 Hz refresh rate) with a black screen background. The subjects were placed approximately 70 cm away from the screen. At the beginning of the task, a fixation cross was displayed at the center of the screen for a random duration between 800-1200ms. This was followed by a search array, including ten circles evenly distributed on the screen, each of which contains a horizontal or vertical line. The search array was presented for 100ms. Participants were instructed to locate the yellow circles within this array and determine the direction of the lines within each yellow circle, all within 100ms. During the experiment, red circles appeared as task-irrelevant stimuli in some trials. The target and distractor were restricted to four positions, located laterally above and below the central gaze point (midline position) on both sides of the central gaze point, and below the horizontal line. The positions of the distractor and target changed under the following conditions: Midline Target No distractor (11.1%), Lateral Target No distractor (22.2%), Lateral Target Midline Distractor (LTMD) (16.6%), Midline Target Lateral Distractor (MTLD) (16.6%), Lateral Target Lateral Distractor Contralateral (LTLD_contra) (16.6%), and Lateral Target Lateral Distractor Distractor Ipsilateral (LTLD_ipsi) (16.6%). The experiment consisted of 24 blocks, each containing 36 trials, for a total of 864 trials. The overall duration of the experiment was 30–40 min. Please refer to Fig. 1.

**Fig. 2:**
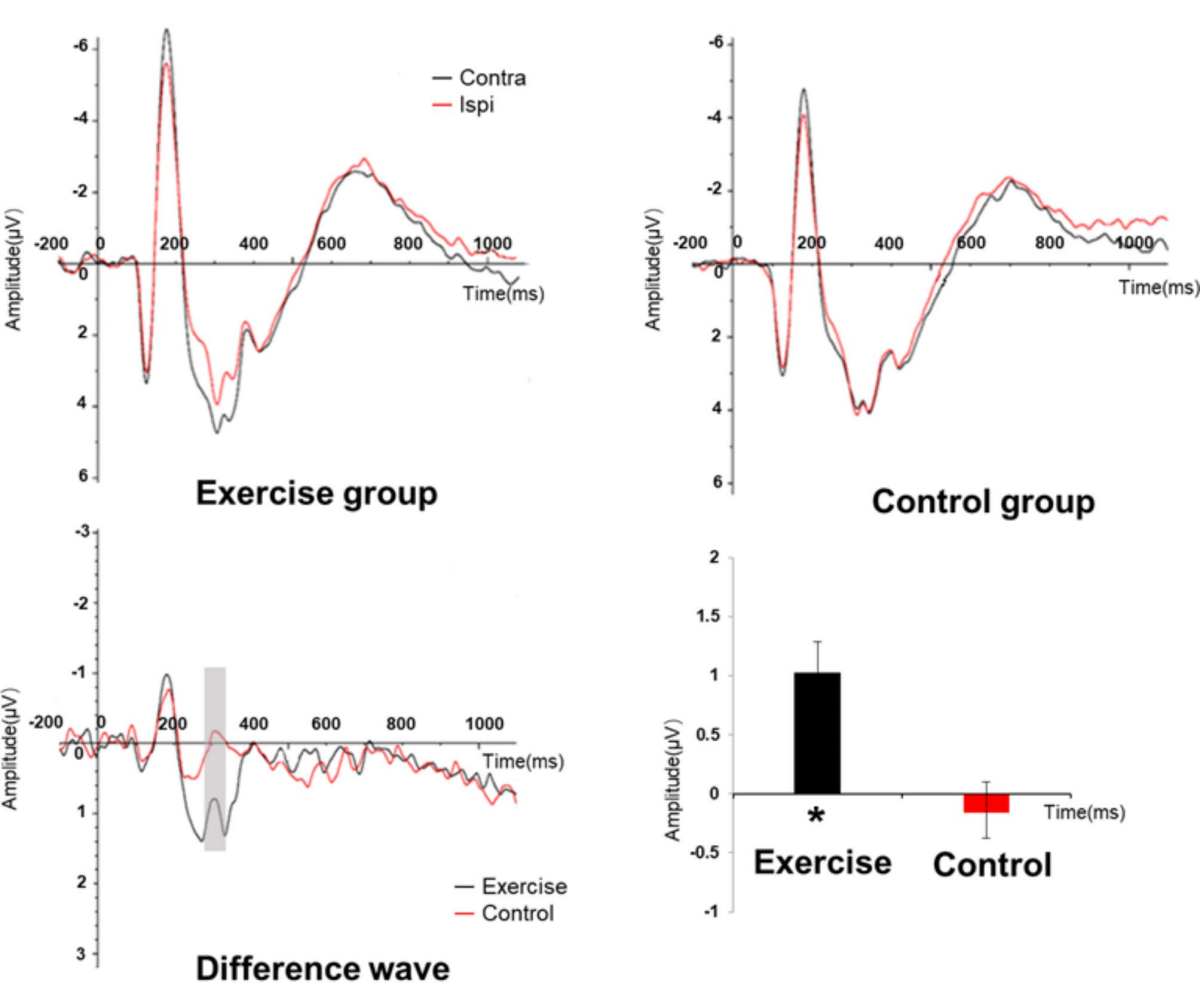
Pd Amplitude of valid condition in experiment1

The visual search task uses E-prime 2.0 software to present the stimuli. The visual search array consists of ten equidistantly presented groups of circles. The distance from each circle to the central point of gaze is 6°, the diameter of each circle is 3.4°, and the width of the border is 0.3°.The circles are colored yellow (77,83,14) red (151,5,9), and green(0,94,11), the lines within the circles are gray (71,81,93),with a black background, all of which have been processed in terms of color brightness to ensure their visual consistency. The yellow circle is the target object, the red circle is a task-irrelevant distraction, and the green circle is a non-target object.

### ERP recording

Using the BrainAmp EEG recording system from Brain Products (Munich, Germany), brainwave data was recorded with a 64-channel Ag/AgCl electrode cap following the international 10–20 system. During online recording, FCz was used as the reference electrode, while offline analysis referenced the bilateral mastoids. Eye movement was recorded using unilateral electrodes, with a single eye electrode placed below the right orbit. The impedance between all electrodes and the scalp was kept below 5 kΩ, and the sampling frequency was set at 1000 Hz.

### Statistical Analysis of Behavioral Data

In the change perception task, the behavioral data of each subject were processed separately as follows: in the test of visual working memory capacity, the percentage of correct responses from each participant was collected under different numbers of stimuli conditions, using the formula: K = S × (H - F), where K represents the individual’s working memory capacity, S represents the number of stimulus arrays, H represents the hit rate (i.e., the percentage of correct judgments when a change occurs), and F represents the probability that the subject misjudged when no change occurred. An independent samples t-test was used to compare whether there were any differences between different groups.

When conducting a visual search task, any responses that are considered incorrect or fall outside a predetermined duration range (either too short or too long) are eliminated from the dataset. After these processing steps, the rejection rate varied between 0.1157% and 7.29%, with an average rate of 1.39%. The remaining trials were classified in two ways: ① by calculating the reaction time and accuracy rate based on the trial type and performing a repeated-measures ANOVA of 2 (group: exercise Group/control group) × 2 (trial type: with/without distractors). ② by calculating the response time and accuracy rate based on the distance between the target and the interferer (the number of non-target distances between the distractor and the target) and performing a repeated-measures ANOVA of 2 (group: exercise group/control group) × 5 (distances: 0, 1, 2, 3, 4). The group was the between-subjects variable, while the trial type and distance were the within-subjects variables. During data analysis, statistics that failed the sphericity test were adjusted for degrees of freedom and p-values using the Greenhouse Gessier method. Post-hoc tests were conducted using the Bonferroni method (See Table 2).

**Table 2:** Basic conditions of the two groups of subjects on the scale scores for experiment 2

Group	Trait Anxiety Score	Baker Depression Score	Visual working memory capacity	Physical activity level (MET-min/W)
Exercise group	54.44 ± 5.575	12.67 ± 6.287	2.42 ± 0.161	576.50 ± 29.21
Control group	54.95 ± 7.382	15.63 ± 8.001	2.57 ± 0.192	587.89 ± 28.33

*Note: The independent sample t-test showed that the difference in trait anxiety scores*,* Baker depression scores*,* visual working memory capacity and physical activity water was not significant*

### EEG data analysis

Offline analysis was conducted using the Matlab EEGlab toolbox and ERPlab. After importing the data, scalp localization was performed for each channel, with the average of the bilateral mastoids used as the reference. The sampling rate was reduced from 1000 Hz to 500 Hz, and filtering was applied within the range of 0.1–30 Hz. Using independent component analysis (ICA) to remove artifacts such as eye movements, muscle activity, cardiac activity, and power line interference from the EEG signals. The EEG signals were segmented according to event types, with a time window from 200 ms before stimulus onset to 800 ms after stimulus onset used for analysis, and a baseline period of 200 ms before stimulus onset. Only responses with correct answers were included in the analysis. Artifacts exceeding ± 80 µV were removed after segmentation, resulting in artifact rejection rates ranging from 0.1 to 18.2%, with an average rejection rate of 2.01%.

For each participant, ERPs to the search displays were collapsed across left and right visual hemifields, as well as left and right electrodes, to produce waveforms recorded contralaterally and ipsilaterally to a lateralized singleton. Based on previous studies, the difference in lateralized ERP was determined by subtracting contralateral and ipsilateral channels using PO7/PO8 [47, 48]. For instance, in experiment 2, in the lateral-target/distractor condition, the target and distractor were displayed on opposite sides. If the target was presented on the left, PO7 represented the contralateral waveform for the left-brain area, while PO8 represented the activity in the right brain area for the target. The Pd were calculated by subtracting PO7 from PO8. The time window chosen for this study is determined based on the peak latency in the average waveform, set at 250ms to 300ms after the presentation of the target stimulus [47, 49, 50]. The time windows of experiment 2 for each condition are presented in Table 2. In experiment1, we performed EEG analysis on valid trials. In experiment2, since the midline object did not elicit the eccentricity-related ERP component, we performed EEG analysis on four positional conditions: lateral target with midline distractor, midline target with lateral distractor, lateral target with ipsilateral distractor, and lateral target with opposite distractor.

## Results

### Behavioral results

#### Experiment 1

The repeated measures ANOVA revealed a significant main effect of stimulus type for response time (F _(2,72)_ = 21.67, *p* < 0.001, η^2^ = 0.568) and accuracy (F _(2,72)_ = 30.26, *p* < 0.001, η^2^ = 0.701), indicating that participants performed significantly more accurately in the neutral cue condition compared to the valid and invalid cue conditions. However, the main effect of group was not significant for response time (F _(1,36)_ = 0.70, *p* = 0.410, η^2^ = 0.020) or accuracy (F _(1,36)_ = 0.26, *p* = 0.613, η^2^ = 0.011), Additionally, there was no significant interaction between stimulus type and group for response time (F _(2,72)_ = 0.04, *p* = 0.828, η^2^ = 0.002) nor accuracy (F _(2,72)_ = 0.21, *p* = 0.609, η^2^ = 0.010) (See Table 3).

**Table 3:** Descriptive statistics of behavioral outcomes for experiment 1 (M ± SD)

		Valid	Neutral	Invalid
Response Time	Exercise group	251.84 ± 90.28	226.35 ± 69.21	255.25 ± 78.80
	Control group	272.53 ± 82.33	249.36 ± 70.45	274.63 ± 76.65
Accuracy	Exercise group	0.92 ± 0.06	0.98 ± 0.02	0.95 ± 0.04
	Control group	0.92 ± 0.07	0.97 ± 0.03	0.97 ± 0.06

### Experiment 2

Descriptive statistics for response time and accuracy were calculated, with the distance between the distractor and target as the within-subject variable and group as the between-subject variable. The results are summarized in Table 4.

A 2 (group)×2 (trial type) repeated measures ANOVA was conducted. The analysis revealed a significant main effect of trial type on response time (F _(1,36)_ = 15.60, *p* < 0.001, η^2^ = 0.308), indicating that participants responded significantly faster in the no-interference condition (560 ms) compared to the interference condition (568 ms). However, the main effect of group was not significant, nor was the interaction between group and trial type. For accuracy, there were no significant main effects for trial type (F _(1,36)_ = 0.06, *p* = 0.808, η^2^ = 0.002), group (F _(1,36)_ = 0.95, *p* = 0.337, η^2^ = 0.026) or their interaction (F _(1,36)_ = 1.25, *p* = 0.271, η^2^ = 0.035).

To account for individual differences in speed-accuracy trade-offs, an independent samples t-test was conducted using the response time/accuracy ratio. The exercise group demonstrated numerically lower values than the control group in both the no-interference (607 vs. 619) and interference conditions (623 vs. 625), though these differences did not reach statistical significance (no interference: t _(36)_ = -0.43, *p* = 0.67, Cohen’s d = 0.14; with interference: t _(36)_ = -0.08, *p* = 0.936, Cohen’s d = 0.02).

**Table 4:** Descriptive statistics of behavioral outcomes for experiment 2 (M ± SD)

		Exercise group		Control group	
		Response time	Accuracy	Response time	Accuracy
distance	0	579.41 ± 76.13	0.92 ± 0.04	566.34 ± 72.78	0.91 ± 0.05
	1	581.71 ± 75.80	0.91 ± 0.04	577.14 ± 77.90	0.89 ± 0.06
	2	568.51 ± 73.20	0.92 ± 0.04	562.15 ± 73.53	0.89 ± 0.06
	3	566.76 ± 83.85	0.92 ± 0.05	551.11 ± 71.31	0.92 ± 0.04
	4	560.88 ± 71.32	0.93 ± 0.04	522.2 ± 67.99	0.92 ± 0.05
distractor	Without	561.94 ± 72.17	0.92 ± 0.04	558.36 ± 71.59	0.91 ± 0.05
	With	572.08 ± 74.25	0.92 ± 0.04	564.02 ± 71.55	0.91 ± 0.05

The effects of the distance between the distractor and the target on response time and accuracy were examined across different interventions. A 2 (group)×5 (target-distractor distance) repeated measures ANOVA revealed a significant main effect of distance on response time (F _(4,144)_ = 11.74, *p* < 0.001, η^2^ = 0.251), response times at contralateral distances 3 and 4 (558 ms, 556 ms) were significantly faster than at the ipsilateral distance 0 (572 ms). However, the main effect of group was not significant (F _(1,36)_ = 0.16, *p* = 0.689, η^2^ = 0.005), and the group×distance interaction was also non-significant (F _(4,144)_ = 0.69, *p* = 0.603, η^2^ = 0.019).

For accuracy, the main effect of distance was significant (F _(4,144)_ = 3.55, *p* = 0.017, η^2^ = 0.307), with accuracy at distance 1 (89.6%) significantly lower than at distances 3 and 4 (92.3% each). The main effect of group was not significant (F _(1,36)_ = 0.95, *p* = 0.337, η^2^ = 0.026). However, a significant interaction between distance and group was observed (F _(4,144)_ = 2.90, *p* = 0.037, η^2^ = 0.266).

### EEG results

#### Experiment 1

The ANOVA results show no significant main effect of stimulus type (F _(2,72)_ = 3.68, *p* = 0.031, η^2^ = 0.093). Main effect of group was significant (F _(1,36)_ = 5.04, *p* = 0.031, η^2^ = 0.123), suggesting a larger Pd amplitude of exercise group (0.39 ± 0.15) than control group(-0.07 ± 0.15). However, there was significant interaction between group and stimulus type (F _(2,72)_ = 4.36, *p* = 0.016, η^2^ = 0.108), showed that the exercise group induced significantly larger Pd components than the control group in valid (F _(1,36)_ = 8.75, *p* = 0.005, η^2^ = 0.196), but not in neutral (F _(1,36)_ = 1.01, *p* = 0.322, η^2^ = 0.027) conditions and the invalid condition (F _(1,36)_ = 0.18, *p* = 0.860, η^2^ = 0.001), suggesting that the exercise group exhibited inhibition of irrelevant stimuli, while the reading group did not. In the exercise group, there was significant difference between three conditions (F_(2,36)_ = 6.95, *p* = 0.003, η^2^ = 0.279). However, no significant difference was found in the control group (F_(2,36)_ = 0.166, *p* = 0.848, η^2^ = 0.009) (See Fig. 2).

#### Experiment 2

**Fig. 3:**
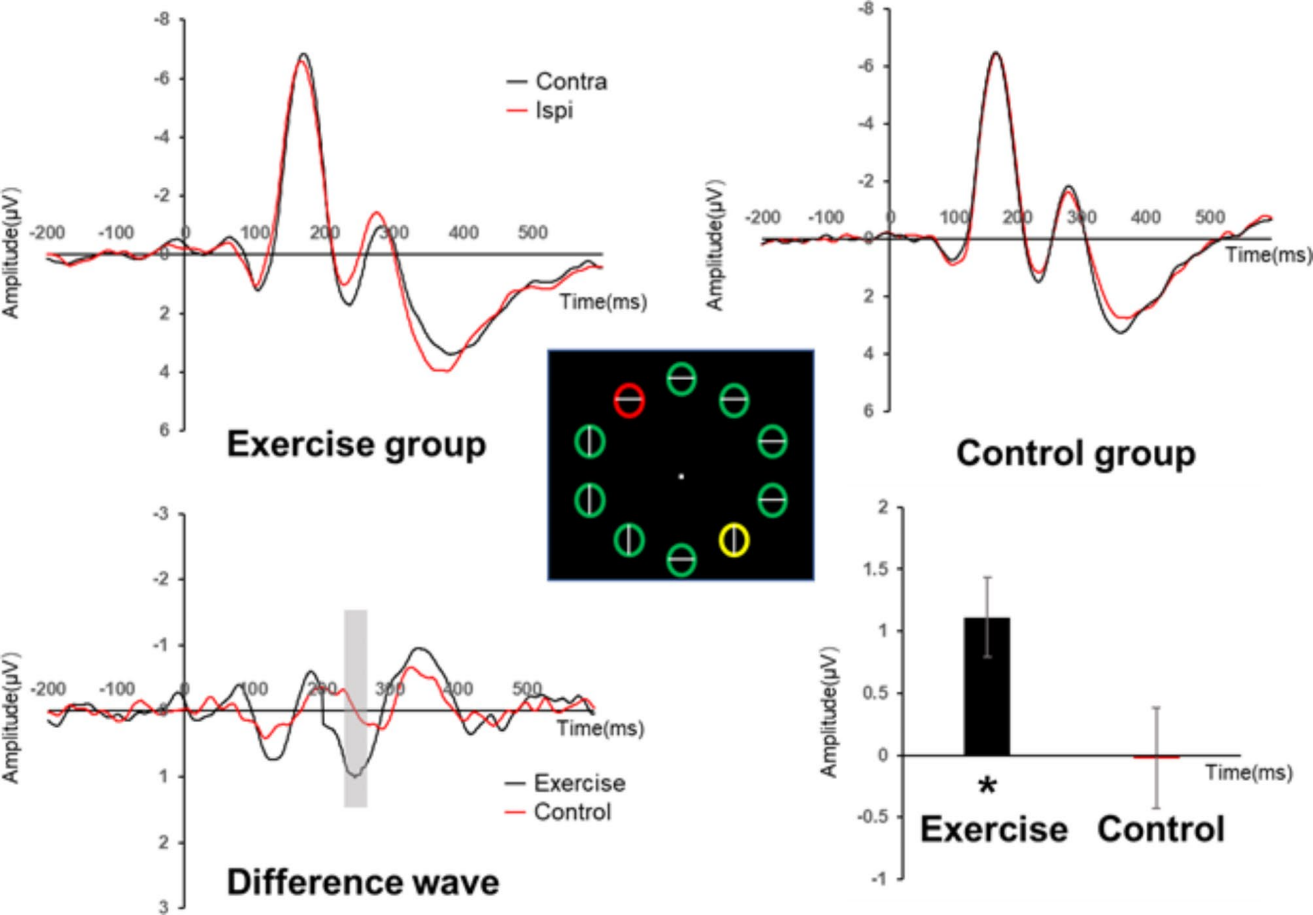
Pd Amplitude of Lateral Target Lateral Distractor Contralateral (LTLD_contra) condition in experiment2

The ANOVA results show no significant main effect of stimulus type (F _(3,108)_ = 2.64, *p* = 0.053, η^2^ = 0.068). Main effect of group was significant (F _(1,36)_ = 6.60, *p* = 0.014, η^2^ = 0.155), suggesting a larger Pd amplitude of exercise group (0.744 ± 0.166) than control group (0.142 ± 0.166). However, there was significant interaction between group and stimulus type (F _(3,108)_ = 3.13, *p* = 0.029, η^2^ = 0.080), revealed that the Pd amplitude was significantly greater in the exercise group than in the control group at the contralateral distribution of target and distractor (F _(1,36)_ = 10.38, *p* = 0.003, η^2^ = 0.224). whereas the mean Pd amplitude was not significant of the exercise group than the control group in the Lateral Distractor Midline Distractor condition (F _(1,36)_ = 2.02, *p* = 0.164, η^2^ = 0.053), and Target Distractor Ipsilateral (F _(1,36)_ = 0.40, *p* = 0.532, η^2^ = 0.011), Midline Target Lateral Distractor (F _(1,36)_ = 0.12, *p* = 0.731, η^2^ = 0.003). This result may reflect the fact that the laterally distributed distractors attracted the attention of subjects in the exercise group. In the exercise group, there was significant difference between conditions (F_(2,36)_ = 3.934, *p* = 0.013, η^2^ = 0.172). However, no significant difference was found in the control group (F_(2,36)_ = 1.343, *p* = 0.271, η^2^ = 0.073) (See Fig. 3).

## Discussion

The present study was designed to explore whether exercise impacts top-down attentional processing in individuals with high-trait anxiety. According to attentional control models, top-down processing involves the active and purposeful selection and allocation of attentional resources to effectively manage perceptual inputs [8]. To explore this, we conducted two event-related potential (ERP) experiments to determine if exercise interventions can enhance the top-down attentional capabilities of individuals with high trait anxiety. A focal ERP component of interest is the Pd wave, which serves as a well-established marker of top-down attentional suppression. The Pd component is characterized by a positive deflection observed at the contralateral electrode compared to the ipsilateral electrode [37, 48, 49]. An increase in the amplitude of the Pd component typically reflects heightened cognitive control demands and the allocation of attentional resources for task completion. Functionally, the Pd component is implicated in directing attention toward task-relevant stimuli while simultaneously inhibiting potential distractors, thereby highlighting its significance in the regulation of attention and cognitive processing in anxiety contexts.

The first experiment comprised three distinct conditions. In the valid cue condition where the target stimulus emerged at the cued location, participants actively directed their attention to the corresponding position and maintained their attentional focus on it. This process is what we refer to as the attention orientation process. The invalid cue condition, on the other hand, was defined by the target stimulus presenting at a different location from the cue. Here, attentional mechanisms entailed not only orientation but also the subsequent shifting of attention. Lastly, the neutral cue condition involved cues that bore no relation to the target’s appearance, and thus, no specific attentional processes were elicited. Significant disparities in Pd amplitudes among different groups were detected specifically in the valid cue condition. This finding implies that exercise intervention had a facilitatory effect on attentional orienting in individuals with high trait anxiety, affording them the ability to detect cues more expeditiously. In contrast, no notable differences were discerned for the invalid cue condition. This lack of significance is plausibly attributable to the intricate nature of the processes involved, as they encompassed both the orientation and subsequent shifting of attention, which might have confounded the detection of distinct effects. Future research endeavors should be directed towards decomposing these complex processes to attain a more lucid understanding of the underlying attentional dynamics.

In Experiment 2, a notable finding emerged where in the Pd amplitude corresponding to distractors in the exercise group exhibited a statistically significant elevation compared to that in the control group. The contrast was especially significant when the target was positioned on the contralateral side relative to the distractors. Simultaneously, the exercise group exhibited reduced response times, which suggests better processing efficiency. In the framework of the visual search paradigm, it is well-established that maximizing the spatial separation between distractors and targets augments the discrimination in their cognitive processing streams [32–34]. It is well-known that high trait anxiety can impair attentional inhibitory ability(Hu, Tang, & Huang, 2023). In the context of this experiment, this means that individuals with high trait anxiety may have more difficulty suppressing the processing of distractors, especially in complex or less spatially distinct situations. However, in the more facilitative contralateral distribution state, where the spatial separation between distractors and targets is maximized, the difference in attentional suppression ability between groups becomes more prominent. This is because in such a state, the normal processing advantage is enhanced, and the negative impact of high trait anxiety on attentional inhibition in the non-exercise group is more likely to be manifested, making it easier to observe the differences between groups. The contralateral distribution configuration accentuated the manifestation of top-down attentional processing, this effect was characterized not only by noticeable behavioral differences but also by a unique Pd amplitude pattern connected to distractors.The overall evidence indicates that an attentional suppression mechanism functions when targets and distractors are placed contralaterally. The results, therefore, imply that participation in moderate-intensity aerobic exercise engendered an augmentation in inhibitory control processes among individuals with high trait anxiety, enhancing their ability to actively filter out irrelevant stimuli. This result aligns with previous studies that have demonstrated the efficacy of exercise interventions in enhancing individuals’ proficiency in handling interference from extraneous stimuli, consequently enabling more effective control of attention [51, 52].This finding supports the idea that exercise is an effective method for strengthening cognitive abilities in people with high levels of trait anxiety, also highlights the particular benefits of exercise in enhancing attentional inhibition. Our research sheds new light on the mechanisms by which exercise interventions can alleviate cognitive impairments associated with high trait anxiety through targeted investigative measures.

Our experimental results are highly consistent with the attention allocation theory, which posits that attentional resources are limited. When performing cognitive tasks, individuals may frequently withdraw attention from one task and reallocate it to another to meet different cognitive demands [53, 54]. Including actively allocating attentional resources to target objects and actively halting and inhibiting the processing of irrelevant information [55, 56].However, despite previous studies clearly demonstrating the role of exercise interventions in enhancing attention, our research further elucidates the mechanisms underlying this effect. Exercise interventions significantly improve the ability to allocate attention to target objects and actively inhibit interference from irrelevant stimuli. The observed improvements in attentional inhibition may extend to other forms of anxiety disorders and emotional conditions characterized by deficits in top-down attentional control. This highlights the potential of exercise interventions as a generalizable strategy to address cognitive impairments associated with a range of emotional and attentional dysfunctions, paving the way for targeted therapeutic approaches.

However, Human cognitive processing encompasses both bottom-up, stimulus-driven processing, and top-down, goal-directed processing. In our study, the behavioral results indicated that the response time for ipsilateral distribution was significantly longer than that for contralateral distribution; however, no significant difference was observed in ipsilateral Pd. These findings suggest the possibility of additional mechanisms influencing the effects of exercise intervention. Previous research has also identified differences in the bottom-up attention processing by testing N2pc component among individuals with anxiety disorders, showing that anxiety also hurt the bottom-up attention processing of individuals [11, 57]. Anxiety is thought to disrupt this delicate balance, impacting both stages of processing [58, 59]. Given these findings, further research could delve deeper into investigating the effects of exercise interventions on various cognitive processing stages. Understanding how exercise impacts both bottom-up and top-down processing in individuals with anxiety disorders could provide valuable insights into potential therapeutic interventions.

For Experiment1, the repeated measures ANOVA revealed a significant main effect of stimulus type on response time and accuracy, with participants performing faster and more accurately in the neutral cue condition compared to valid and invalid cue conditions. This aligns with previous research suggesting that neutral cues may facilitate response speed by avoiding directional or attentional demands [60].However, the main effect of group was not significant for either response time or accuracy, indicating no observable differences between the exercise and reading groups. It is possible that the sample size or the specific characteristics of our participant groups did not allow for the detection of more subtle differences, or that the effects of exercise and reading on response time may be more complex and require further investigation with more refined measures or experimental designs. This suggests that the effects of exercise and reading on task performance may be more nuanced, potentially influenced by unmeasured factors or requiring larger sample sizes for detection. The absence of significant interaction effects further supports the idea that both groups responded similarly to different stimulus types, highlighting the complexity of exercise’s influence on attentional processes in this paradigm.The results indicates that the effect of stimulus type on response time is similar across the exercise and reading groups. In other words, both groups responded to the different stimulus types in a comparable manner, without showing any differential sensitivity or adaptation based on their group membership. This finding further supports the idea that, at least in terms of the current experimental paradigm and measured variables, the influence of exercise and reading on response time and accuracy may not be as straightforward as initially hypothesized, and other factors may be involved in modulating the relationship.

For Experiment2,the analysis revealed a significant main effect of trial type on response time. This finding is in line with our expectations and supports the theoretical understanding that interference can impede response speed, as participants likely had to allocate more cognitive resources to handle the interference, thus resulting in slower responses. However, the main effect of group was not significant, nor was the interaction between group and trial type. This could imply that, within the context of our study, the factor that differentiates the groups (e.g., the specific characteristics or experiences related to the exercise group and the control group) did not have a substantial impact on the general response time. It is possible that other unmeasured or uncontrolled factors may have masked any potential group differences, or that the sample size was not sufficient to detect more subtle effects. Future research could explore additional variables or increase the sample size to further investigate potential group differences.In terms of task accuracy, neither the trial type nor the group membership had a significant influence. It is possible that the task was not sufficiently challenging or sensitive to detect differences in accuracy, or that the participants’ performance was relatively consistent across different conditions and groups. Additionally, individual differences in cognitive abilities or strategies may have contributed to the lack of significant effects, and future studies could consider incorporating measures of individual differences to better understand the sources of variation in accuracy.

An independent samples t-test for response time/accuracy ratio showed numerically lower ratios in the exercise group, suggesting more efficient task performance, as participants responded faster while maintaining higher accuracy.The non-significant results of the t-test suggest that, despite the observed numerical differences, we cannot confidently conclude that there is a true difference in the efficiency of task performance between the two groups based on the response time/accuracy ratio. However, it is important to note that the small effect sizes (Cohen’s d) indicate that the differences, if any, are relatively small. This could be due to factors such as limited sample size, measurement error, or the presence of other confounding variables that may have influenced the relationship between response time and accuracy. Future research could aim to increase the sample size, improve the measurement precision, and control for potential confounding factors to more accurately assess the potential differences in speed-accuracy trade-offs between the groups.

The current study adopts a cross-sectional design, with the aim of assessing the immediate impacts of a single session of aerobic exercise. Nevertheless, should a longitudinal design be utilized to capture the effects of repeated or continuous exercise, it is likely that the findings would reveal more pronounced and enduring alterations in cognitive function. As previous research has indicated, chronic exercise has the potential to augment neuroplasticity, especially within regions associated with cognitive control, such as the prefrontal cortex and hippocampus [44, 61]. This particular design would be conducive to establishing causal associations between exercise and the enhancement of attention control over an extended period, thereby presenting a more distinct and comprehensive understanding of the long-term advantages of exercise for individuals with high trait anxiety.

The implementation of repeated exercise interventions could further emphasize the cumulative advantages of exercise on cognitive function. It has been demonstrated by numerous studies that regular aerobic exercise conducted over several weeks or months can exert a substantial influence on executive function and attentional control [62]. Consequently, this might lead to more substantial and persistent modifications in EEG indicators, for instance, the Pd component, which serves as an indication of enhanced inhibitory control. Additionally, repeated exercise sessions may also disclose behavioral changes that were not observable in a single-session study design.

A key factor to consider in future research is the intensity of exercise. Different intensities can have varying impacts on cognitive processes and neural mechanisms. For example, moderate to vigorous aerobic exercise has been shown to improve attention and executive function more effectively than low-intensity exercise [63, 64]. Including a range of exercise intensities could help delineate optimal conditions for cognitive enhancement in anxious individuals, allowing for more targeted exercise prescriptions. Comprehending the long-term cognitive effects of exercise is of utmost importance for the development of sustainable interventions targeting high trait anxiety. Longitudinal studies have indicated that sustained aerobic exercise can bring about lasting improvements in cognitive flexibility and inhibitory control, accompanied by structural brain changes such as an increase in gray matter volume within prefrontal areas [65, 66]. Future research endeavors could center on exploring whether long-term exercise not only influences neural markers but also translates into tangible real-world behavioral changes related to attentional control and anxiety management.

A limitation of the current study is the absence of a low trait-anxious comparison group. Without such a group, it is difficult to ascertain whether the observed differences in the effects of exercise intervention are specific to individuals with high trait anxiety or are reflective of general effects that might also apply to individuals with lower levels of anxiety. This limitation restricts our ability to generalize the findings across different levels of trait anxiety.Future studies should aim to include both high and low trait-anxious participants to provide a comprehensive understanding of how trait anxiety interacts with exercise interventions. Such an approach would help determine whether the benefits of exercise are uniquely pronounced in high-anxiety populations or whether they extend across a broader range of anxiety levels. Additionally, this would allow for a more nuanced exploration of the mechanisms underlying these effects and inform tailored intervention strategies for individuals with varying levels of anxiety.

Based on the findings of this study, a promising direction for future research is to explore the potential of aerobic exercise in enhancing attentional control in aging populations, particularly in individuals with high trait anxiety. Drawing upon the Compensation-Related Utilization of Neural Circuits Hypothesis (CRUNCH) proposed in aging research, it is suggested that older adults might utilize additional neural circuits to compensate for age-related cognitive decline [67]. Similarly, individuals with high trait anxiety may engage extra neural resources to counteract the cognitive impairments caused by anxiety. Combining these two concepts, future studies could investigate whether exercise can enhance the compensatory neural processes in older adults, similarly to how it has been shown to improve cognitive control in individuals with high trait anxiety. By examining the neural mechanisms behind these compensatory responses, researchers could better understand how exercise interventions might support cognitive functions in aging populations and offer strategies to mitigate the cognitive effects of both aging and anxiety.

In conclusion, our research not only highlights the beneficial effects of exercise on the enhancement of top-down attentional abilities but also provides evidence supporting the impact of moderate-intensity acute aerobic exercise on executive functions. These findings suggest that even a single session of moderate-intensity aerobic activity can facilitate cognitive improvements, which is particularly relevant for individuals with anxiety disorders. Moreover, our results set the stage for future investigations into the nuanced effects of varying exercise intensities and frequencies on executive function enhancement within this population. By exploring these dimensions more deeply, subsequent studies can further elucidate how tailored exercise interventions may be optimized to improve cognitive outcomes for individuals suffering from anxiety, thereby informing therapeutic strategies aimed at enhancing overall mental health and functioning.

## Data Availability

Data availability Data is openly available in a public repository at https://osf.io/8cd93/.
